# VX765 Attenuates Pyroptosis and HMGB1/TLR4/NF-*κ*B Pathways to Improve Functional Outcomes in TBI Mice

**DOI:** 10.1155/2020/7879629

**Published:** 2020-04-15

**Authors:** Zhezhe Sun, Mark Nyanzu, Su Yang, Xiaohong Zhu, Kankai Wang, Junnan Ru, Enxing Yu, Hengli Zhang, Zhenzhong Wang, Jie Shen, Qichuan Zhuge, Lijie Huang

**Affiliations:** ^1^Department of Neurosurgery, The First Affiliated Hospital of Wenzhou Medical University, Wenzhou 325000, China; ^2^Zhejiang Provincial Key Laboratory of Aging and Neurological Disorder Research, The First Affiliated Hospital of Wenzhou Medical University, Wenzhou 325000, China; ^3^Department of Neurosurgery, Yuyao People's Hospital, Ningbo 315000, China; ^4^Department of Neurosurgery, The First Affiliated Hospital of Medical School of Zhejiang University, Hangzhou 310003, China

## Abstract

**Background:**

Traumatic brain injury (TBI) refers to temporary or permanent damage to brain function caused by penetrating objects or blunt force trauma. TBI activates inflammasome-mediated pathways and other cell death pathways to remove inactive and damaged cells, however, they are also harmful to the central nervous system. The newly discovered cell death pattern termed pyroptosis has become an area of interest. It mainly relies on caspase-1-mediated pathways, leading to cell death.

**Methods:**

Our research focus is VX765, a known caspase-1 inhibitor which may offer neuroprotection after the process of TBI. We established a controlled cortical impact (CCI) mouse model and then controlled the degree of pyroptosis in TBI with VX765. The effects of caspase-1 inhibition on inflammatory response, pyroptosis, blood-brain barrier (BBB), apoptosis, and microglia activation, in addition to neurological deficits, were investigated.

**Results:**

We found that TBI led to NOD-like receptors (NLRs) as well as absent in melanoma 2 (AIM2) inflammasome-mediated pyroptosis in the damaged cerebral cortex. VX765 curbed the expressions of indispensable inflammatory subunits (caspase-1 as well as key downstream proinflammatory cytokines such as interleukin- (IL-) 1*β* and IL-18). It also inhibited gasdermin D (GSDMD) cleavage and apoptosis-associated spot-like protein (ASC) oligomerization in the injured cortex. In addition to the above, VX765 also inhibited the inflammatory activity of the high-mobility cassette -1/Toll-like receptor 4/nuclear factor-kappa B (HMGB1/TLR4/NF-kappa B) pathway. By inhibiting pyroptosis and inflammatory mediator expression, we demonstrated that VX765 can decrease blood-brain barrier (BBB) leakage, apoptosis, and microglia polarization to exhibit its neuroprotective effects.

**Conclusion:**

In conclusion, VX765 can counteract neurological damage after TBI by reducing pyroptosis and HMGB1/TLR4/NF-*κ*B pathway activities. VX765 may have a good therapeutic effect on TBI.

## 1. Background

Traumatic brain injury (TBI) is a physical brain injury caused by blunt mechanical force [1], characterized by high mortality and morbidity. However, the clinical conversion of drug therapy in TBI patients is still insufficient [2]. The rising incidence of TBI is related to the acceleration of urbanization, the increase of traffic accidents, and the frequency of local wars. According to data, TBI will become the main cause of diseases by 2010 [3]. TBI pathophysiology involves major mechanical damage and multiple secondary damage cascades (e.g., oxidative stress, apoptosis, and neuroinflammation) [4]. A growing body of evidence shows that innate immunity and neuroinflammation are connected with TBI pathogenesis [5]. Specifically, the inflammatory response may be an early and prominent pathological feature of TBI [6–8]. In addition to regional inflammatory responses in microglia and nerve cells, damage will also irritate the invasion of macrophages and neutrophils; as a result, it leads to the release of a large number of proinflammatory factors. Interleukin- (IL-) 1*β* produced in the course is well noted. It provides explicit proof for the major role of this cytokine in TBI-related inflammation [8–10]. Excessive inflammation may further damage the integrity of the blood-brain barrier (BBB) and advance the invasion of more peripheral immune cells [11]. Therefore, proper regulation of neuroinflammation may be a useful approach for TBI.

Pyroptosis can be defined as a highly specific inflammatory programmed cell death. It differs from necrosis or apoptosis [12], which depends on extracellular detection of acute injury to determine extracellular as well as intracellular pathogen-related molecular patterns (PAMPs) of NOD-like receptors (NLRS) or IM2-like receptors (AIM2) in melanoma 2(A). NLR and AIM2 can lead to the formation of multiprotein complexes, called inflammasomes, which contain apoptosis-associated spot-like proteins (ASCs) as well as pro-caspase-1, and this process sends out signals that cause a series of inflammatory reactions [13]. Once pyroptosis is activated, the inflammasome protein complex polymerizes and causes pro-caspase-1 to cleave into proteolytically active subunits. Active caspase-1 cleaves IL-1*β* coupled with IL-18 into active forms and then excretes them into extracellular space [14]. Recently, it was reported that gasdermin D (GSDMD) cleavage and pore formation are essential components of pyroptosis in human cells, and cells that do not express GSDMD undergo apoptotic cell death [15]. Activated GSDMD combines intimal lipid through plasma membrane transport, and then oligomerizes to form membrane pores. As a result, local cell swelling, membrane rupture, and cell extravasation occur [16–20]. Numerous studies have shown that pyroptosis occurs in many neurological conditions and is also involved in the development of atherosclerosis and other systemic diseases [21, 22]. In recent years, it has been found that inflammation-mediated lower eyelid ptosis participates in the pathological development of TBI. In addition, activated inflammatory complexes in cytoplasm are considered a necessary step for neuroinflammation in secondary brain injury [23]. In these inflammatory complexes, NLR and AIM2, particularly, the pyrin domain of NLR family consisting of 1(NLRP1) and NLRP3, play important roles in the occurrence and development of TBI. They can be found in neurons, astrocytes, and microglia in damaged brain tissue, where they accelerate the induction of inflammatory responses and neuronal death, and aggravate neurological results [24]. Toll-like receptor (TLR), a pattern recognition receptor for innate immune responders [25], can be activated by molecular patterns associated with cell damage products [26]. Numerous studies have shown that some TLR subtypes, comprising TLR4, are widely demonstrated in the brain and play important roles in regulating inflammation following brain injury [27, 28]. NF-*κ*B is a main transcription element and can be stimulated by TLR4 [29, 30]. The nucleotide binding and oligomerization domain- (NOD-) like receptor family consists of the pyrin domain 3 (NLRP3) inflammasome which is identified as an essential signaling molecule downstream of TLR4 which can advance the maturation of inflammatory cytokines like IL-1*β* [31, 32]. Therefore, the pyroptosis-associated inflammasome and TLR4/NF-*κ*B pathway are closely linked with the pathogenesis of TB1.

VX765 is regarded as a newly developed and selective small molecule caspase-1 inflammatory inhibitor that reduces inflammation in vitro and in vivo [33, 34]. However, the effects of VX765 treatment on apoptosis, BBB destruction, microglial activation, and nervous system are not explicit. During the process of this study, we implied clinically relevant pharmacological analyses in a mouse TBI model with the aid of VX765. We assumed that neurological outcomes after trauma can be improved by administering VX765 to reduce the inflammatory reaction of TBI.

## 2. Methods

### 2.1. Animals and Grouping

In this study, we purchased adult male C57BL/6 mice (8-10 weeks old, 20-25 g) from the Shanghai Slaccas Experimental Animal Limited Liability Company (Shanghai, China). Under 12 h of light and dark circulation, the mice were placed in animal care facilities and were free to access food and water. The experiment was carried out under the supervision of Wenzhou Medical University. Mice were isolated and reared for 1 week and then randomized into four groups: sham surgery, controlled cortical impact (CCI), CCI+vehicle (DMSO), and CCI+VX765. VX765 (catalog number: HY-13205; MedChemExpress, Monmouth Junction, NJ, USA) dissolved in DMSO was injected intraperitoneally (100 mg/kg) 1 h after CCI and every 2 days after CCI for two weeks [33, 35]. At the same time point, the CCI+vehicle group was given an equal volume of DMSO. In this study, we followed the methods of Xu et al. [36].

### 2.2. CCI Model

Our method for inducing TBI in mice was described previously [37]. We anesthetized animals with intravenous chloral hydrate (5%, 0.1 ml/10 g) and then fixed them in a stereotactic frame (Kopf Instruments, Tujunga, CA, USA). After disinfection with iodophor, the right parietal cortex (2.0 mm posterior to anterior and posterior iliac crest, 2.0 mm lateral sagittal suture) underwent 3.5 mm craniotomy with dura intact. The attack speed of mice in the CCI group was 4.5 m/s for 200 ms, retaining the inhibitory effect of 2.0 mm, and then, moderate TBI with a diameter of 3 mm was produced on the head. After scalp incision, the mice were sutured with discontinuous 4-0 silk thread and finally placed in a heated cage for recovery. The sham group was only performed with right parietal craniotomy. All operations are carried out using rigorous aseptic techniques.

### 2.3. Western Blotting

We microdissected the control cortex from the brain collected from the sacrificed animal and froze it in liquid nitrogen immediately. The tissue sample was then homogenized and lysed in RIPA buffer plus [38] with protease inhibitor mixture for the Western blot (WB) test. Sodium dodecyl sulfate- (SDS-) sodium polyacrylate (SDS) was loaded with the same amount of protein (8 units g per channel). E-Gel electrophoresis as well as electrophoresis was changed to 0.22 *μ*m polyvinylidene fluoride membrane (preactivated with methanol; Millipore, Billerica, MA, USA). After the membranes were blocked with 5% skimmed milk, Tris-buffered saline with tween-20 (TBST) was incubated at room temperature for 2 h, and the membrane was incubated with the corresponding main antibody overnight at 4°C. Then, 8% SDS-PAGE gel was used for detection on NLRP1 (1 : 1000, Abcam), NLRP3 (1 : 1000, CST), NLRC4 (1 : 1000, Abcam), and zona pellucida 1 (ZO-1, 1 : 1000, Invitrogen), and 10% SDS-acrylamide gel was applied for AIM2 (1 : 1000, Abcam), GSDMD (1 : 1000, Abcam), and NF-*κ*B (1 : 1000, CST). Twelve percent SDS-acrylamide gels were used to detect p-I*κ*B*α* (1 : 1000, CST), I*κ*B*α* (1 : 1000, CST), ASC (1 : 1000, CST), caspase-1 (1 : 1000, Proteintech), cleaved-caspase-1 (1 : 1000, CST), IL-1*β* (1 : 1000, Abcam), IL-18 (1 : 1000, Abcam), HMGB1 (1 : 1000, Proteintech), TLR4 (1 : 1000, Proteintech), and cleaved-caspase-3 (1 : 1000, Abcam). Glyceraldehyde 3-phosphate dehydrogenase (GAPDH; 1 : 1000, CST), *α*-tubulin (1 : 1000, Abcam), and *β*-actin (1 : 1000, Abcam) were selected as internal controls as appropriate. After incubation for 24 h, the membranes were rinsed and incubated with proper secondary antibody (1 : 10000) for 1 h. Banding results were quantified with the aid of Image Lab software (Bio-Rad, Hercules, CA, USA), and the target protein signal intensity was compared to GAPDH intensity.

### 2.4. Lactate Dehydrogenase (LDH) Release Detection

Mice were incapacitated by deep anesthesia, and then, blood was collected (3000 g centrifuged for 10 min). After blood collection, the release of serum LDH was measured by a commercial kit (Beyotime, Shanghai, China). After 100 *μ*l of serum or supernatant was transferred to a 96-well plate, the response mixture was put in and incubated in the dark lasting for 30 min at room temperature. LDH concentration was determined through detecting absorption at 490 nm.

### 2.5. Enzyme-Linked Immunosorbent Assay (ELISA)

A bicinchoninic acid (BCA) protein assay kit was used to take a measurement of total protein concentration. The level of transforming growth factor- (TGF-) *β* was gauged with an ELISA (Beyotime, Shanghai, China) according to the manufacturer's instructions.

### 2.6. Caspase-1 Activity Assay

Activated caspase-1 was gauged with the aid of a colorimetric assay (Beyotime, Shanghai, China) according to the manufacturer's protocol. To be brief, the damaged cortex was lysed in ice-cold RIPA buffer (1 mM phenylmethylsulfonyl fluoride (PMSF)) and centrifuged at 2000g and 4°C lasting for 10 minutes. Cortical supernatant was taken and incubated with the aid of acetyl-Tyr-Val-Al-Asp-nitroaniline (Ac-YVAD-PNA) (2 mm) at 37°C lasting for 2 h. Activated caspase-1 was estimated by spectrophotometric measurement of PNA. This substance is cut from the substrate Ac-YVAD-PNA. PNA is a molecular device M2 flat-panel reader at 405 nm (Molecular Devices Company, San Jose, California, United States). Caspase-1 activity was decided by inserting a serial dilution of the standard curve derived from the PNA standard and normalizing to total protein. The protein concentration was determined through the Bradford method to keep the concentration in a gradient of 1-3 *μ*g/*μ*l. Data are regarded as relative caspase-1 activity opposite to the control.

### 2.7. Terminal Deoxynucleotidyl Transferase-Mediated dUTP-Biotin Nick End Labeling (TUNEL) Staining

The cerebral cortex was obtained and sliced to measure apoptosis with the TUNEL Apoptosis Assay Kit (Beyotime, Shanghai, China). TUNEL stained apoptotic nuclei, and 4′,6-diamidino-2-phenylindole (DAPI) with fluorescein-dUTP stained all nuclei. The apoptosis index (AI) is calculated by dividing the number of TUNEL-positive cells by the total number of cells per field of view. AI was assessed in 15 fields chosen at random.

### 2.8. Immunohistochemical Analysis

After the mouse was sacrificed, the brain tissue was removed and then incubated in 4% paraformaldehyde phosphate buffer solution (PBS) immediately and embedded in paraffin for sectioning. After the sections were rehydrated and washed twice in PBS, 3% H_2_O_2_ was applied to block the endogenous peroxidase activity. Antigen crosslinking was carried out in an autoclave lasting for 3 min, after which the sample was cleaned twice in PBS. All preparations were coped with the aid of a goat serum blocking agent (Abcam, ab7481) for 45 min. Then, PBS was used to rinse quickly and remove excess reagent. These parts were incubated with the original antibody overnight. After the unbound antibody was washed, the secondary antibody (ASS3403; Abgent, San Diego, CA, USA) was put in crosslink lasting for 30 min. After that, sections were rinsed and nucleus-stained with DAPI (Solarbio, DA 1010) for 5~10 min. All preparations were then counterstained with the aid of hematoxylin (Solarbio, G1120) and lasted for 30 s. Protein expression was decided in four adjacent parts per sample.

### 2.9. Double Immunofluorescence Staining

Animals were sacrificed after 24 h of TBI treatment followed by cardiac perfusion with 140-180 ml of 0.9% normal saline (heparinized) and subsequently with 140-180 ml of cold 4% paraformaldehyde in PBS (pH 7.5). After that, we carefully collected the brain and immersed it in formaldehyde fixative (10% formaldehyde in 0.1 M sodium phosphate buffer) and 4% paraformaldehyde at once and kept at 4°C for 2 days. Next, the brain tissue was fixed in 30% sucrose until it sank to the bottom of the container. The brain tissue was then removed for embedding in an optimal cutting temperature (OCT) composite frozen section. After incubation with acetone at -20°C for 20 min as well as 3% bovine serum albumin (BSA, Sigma-Aldrich) solution at 37°C lasting for 30 min to block nonspecific staining, the parts were then incubated with the aid of subsequent antibodies at 4°C overnight: Iba-1 (1 : 500, Abcam), caspase-1 (1 : 500, Abcam), iNOS (1 : 500, Abcam), CD206 (1 : 500, Abcam), IL-1*β* (1 : 500, Abcam), and IL-18 (1 : 500, Abcam). The frozen parts were then washed and incubated with corresponding secondary antibodies for 1 h at RT. At last, the nuclei were counterstained with DAPI or TUNEL. Images of each part were collected with the aid of a fluorescence microscope, and data were analyzed based on 15 fields selected at random (5 sections per slice × 3 slices per mouse) under a microscope with the application of ImageJ software.

### 2.10. Coimmunoprecipitation (Co-IP)

After treatment with DMSO or VX765, the supernatant of brain tissue in the CCI group was collected. Then, 400 *μ*l of supernatant was used for the IP assay and incubated with NLRP3 antibody or IgG antibody (negative control) at 4°C overnight. Protein G beads were added for incubation at 4°C for 3~5 h using a rotator. The mixture of the above buffer was centrifuged at 4°C and 1000g for 5 min. Then, the supernatant was removed, and the complex was collected. Following the complex was washing by washing buffer (50 mM Tris-HCl/pH 7.4, 100 mM NaCl, 5 mM CaCl_2_, 5 mM MgCl_2_, 0.1% Nonidet P-40) for three times; the sediment was resuspended with 1x SDS-PAGE loading buffer. Subsequently, the prepared protein samples were subjected to metal bath at 100°C for 5 min, followed by electrophoresis in 10% polyacrylamide gel. The separated proteins were imprinted on PVDF membranes. NLRP3 (ab263899, 1 : 30, Abcam, USA) and ASC (ab151700, 1 : 50, Abcam, USA) were used as primary antibodies, and goat anti-rabbit IgG (1 : 5000, Beijing ComWin Biotech Co., Ltd., Beijing, China) was applied as secondary antibody for Western blotting.

### 2.11. Assessment of Cerebral Edema

Brain water content (BWC) is defined as a sensitive approach in order to measure cerebral edema and quantified with the aid of dry and wet methods, as explained in previous statements [38]. At different time points after injury, mice were sacrificed and brain tissues were quickly removed. Then, we weighed the tissue samples in order to decide the wet weight (WW). We put the sample in an oven and dried it at 100°C for 24 h so as to obtain dry weight (DW). BWC was calculated with the help of the following form: A: 100% × (WW‐DW)/WW.

### 2.12. Measurement of Lesion Volume

In order to quantify the volume of brain damage 14 days after injury, a 120 *μ*m continuous cross-section was cut to cover the entire damaged cortex, as explained above [39]. After staining with hematoxylin and eosin (Beijing, China), sections were then observed and imaged with an optical microscope (Olympus). The ipsilateral as well as contralateral hemisphere volumes were calculated blindly with the help of ImageJ software. Lesion volume was calculated by numerical integration of successive regions, and the results are expressed as the volume percentage of the lesion weighed against the contralateral hemisphere.

### 2.13. Evans Blue Dye Extravasation

Evans Blue (EB) was applied to assess BBB permeability according to the explanation previously described [11]. Briefly, EB solution was injected through the tail vein after 24 h of CCI and circulated lasting for 2 h. After that, reanesthetized mice were perfused with ice-cold PBS so as to remove intravascular EB dye. After that, they were sacrificed at once. The hemispheres were taken for dissection, weighing, and homogenization in N,N-dimethylformamide at 60°C for 72 h, followed by centrifugation at 14000 rpm to collect supernatant. The absorbance of the supernatant at 620 nm was measured for quantification of EB concentration in micrograms per gram of brain tissue.

### 2.14. Neurobehavioral Training and Evaluation

The modified nerve severity score (mNSS) was performed at 12, 24, 48, and 72 h after TBI or sham operation to evaluate the total neurological deficit. The test evaluated the movement, sensation, reflex, and balance capabilities. The mNSS test is divided into 0 level (normal performance) to 18 level (maximum defect). The higher score meant more serious neurological dysfunction. Therefore, mice with abnormally preoperative scores were excluded.

The main test method of rotating rod test measured the ability of mice on a spinning rod in order to evaluate good coordination and balance of motion. One day before surgery, the mice were primarily subjected to an adaptive test (rotation speed: 4 rpm), and another four tests were performed to accelerate the rotation speed. In the experiment, the average time for the rotating rod to fall off was recorded to get a stable baseline value before injury. At 12, 24, 48, and 72 h postinjury, each mouse carried out testing four times a day at the same rate with an interassay interval of 30 min, and we measured the mean descent latency.

### 2.15. Statistical Analysis

All statistics are based on more than three independent studies. The statistics are demonstrated as the mean ± standard deviation (SD). Analyses of BWC test data and ELISA at different time points were performed with the aid of two-way analysis of variance (ANOVA). It was followed by least obvious difference (LSD) post hoc analysis. For other statistics, one-way ANOVA was used, followed by LSD post hoc analysis or Student's *t*-tests to analyze statistical comparisons. *P* < 0.05 was expressed as significant.

## 3. Results

### 3.1. The Effects of Caspase-1 Inhibitor VX765 on IL-1*β* and IL-18 Levels

**Figure 1:**
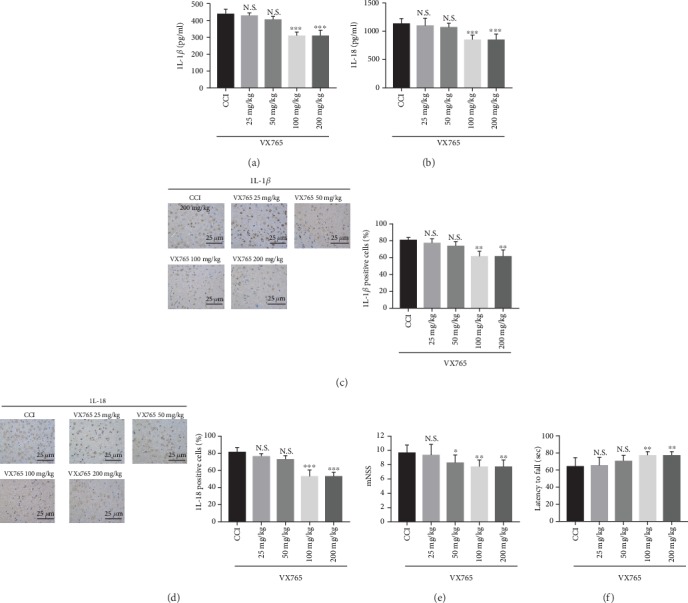
The effects of different concentrations of the caspase-1 inhibitor VX765 on IL-1*β* and IL-18 levels in CCI and VX765 groups. The levels of (a, c) IL-1*β* and (b, d) IL-18 in the injured cortex were measured by ELISA and immunohistochemistry 24 h after TBI at different concentrations of VX765 (25, 50, 100, or 200 mg/kg). Note that the levels of IL-1*β* and IL-18 were significantly decreased for the concentrations of VX765 (100 and 200 mg/kg). At the lowest concentrations of VX765 (25 and 50 mg/kg), there was no significant change in IL-1*β* or IL-18 compared with the CCI group. Detection on the levels of mNSS (e) and latency to fall (f) through the rotarod test at 24 h after TBI with different concentrations of VX765 (25, 50, 100, or 200 mg/kg) suggested that the mNSS level was decreased and latency to fall was increased in the groups treated with VX765 at the concentration of 50, 100, and 200 mg/kg, and levels of mNSS and latency to fall had no significant difference in the group treated with 25 mg/kg VX765, compared to the CCI group. *n* = 4. Data are presented as the mean ± SD. ^∗∗^*P* < 0.01, ^∗∗∗^*P* < 0.001. N.S.: not significant vs. CCI.

To determine whether VX765 can alleviate inflammatory cytokine levels (IL-1*β* and IL-18) and identify the optimal dose, we examined inflammation around the injured cortex 24 h after CCI mice received different drug concentrations. We found that the expression levels of IL-1*β* (Figure 1(a)) as well as IL-18 (Figure 1(b)) were significantly inhibited by VX765 at concentrations of 100 or 200 mg/kg. However, the concentration at 25 or 50 mg/kg showed no obvious difference. Our results of immunohistochemistry (Figures 1(c) and 1(d)) and behavioral test (Figures 1(e) and 1(f)) also identified the above results. Therefore, VX765 concentration of 100 mg/kg was adopted in subsequent experiments [35].

### 3.2. Expressions of Inflammatory Cytokines in Cortex after TBI

**Figure 2:**
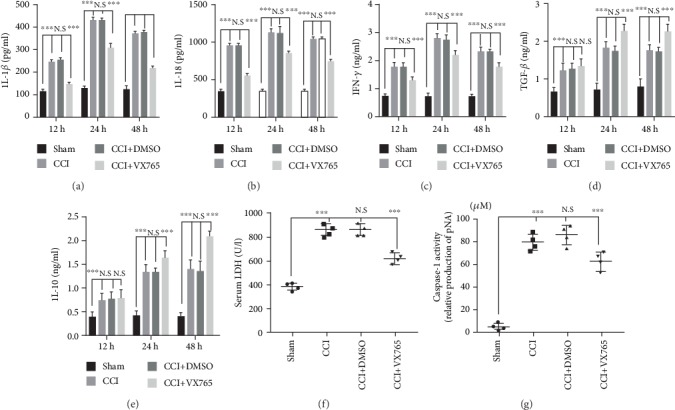
Expression of inflammatory cytokines in the cortex after TBI. Compared with the sham operation group, the concentrations of proinflammatory cytokines (a) IL-1*β*, (b) IL-18, and (c) IFN-*γ* and anti-inflammatory cytokines (d) TGF-*β*1 and (e) IL-10 were significantly increased by ELISA in the injured lesion at 12, 24, and 48 h. Compared with the DMSO group, proinflammatory cytokines in the VX765 group showed significant decreases at three time points after trauma. There was no significant change in anti-inflammatory factors in the VX765 group compared to the DMSO group at 12 h after TBI; however, there was a significant increase in anti-inflammatory factors in the VX765 group at 24 and 48 h after TBI. (f) Serum LDH was tested at 24 h following TBI since cerebral injury increases cellular LDH leakage. There was a significant decrease in serum LDH in the VX765 group. (g) Caspase-1 activity was stimulated after TBI at 24 h. In contrast, the TBI-induced increase in caspase-1 activity was significantly reduced in mice receiving VX765 treatment. *n* = 4. Data are presented as the mean ± SD. ^∗∗^*P* < 0.01, ^∗∗∗^*P* < 0.001. N.S.: not significant.

In order to compare expression levels of inflammatory factors in the cortex, a series of inflammatory cytokines were determined by ELISAs. We found that all detected cytokine levels were lower in the sham group against the other groups (Figure 2). Results showed that the three proinflammatory cytokines IL-1*β* (Figure 2(a)), IL-18 (Figure 2(b)), and IFN-*γ* (Figure 2(c)) were all obviously decreased in the VX765 group, compared to the DMSO group at 12, 24, and 48 h. While at 12 h after TBI, there were no significant changes in anti-inflammatory factors including TGF-*β*1 and IL-10 in the VX765 group compared with the DMSO group. However, those anti-inflammatory factors were significantly increased in the VX765 group at 24 and 48 h after TBI (Figures 2(d) and 2(e)), suggesting that VX765 administration effectively decreased secretion of inflammatory cytokines. LDH is essential for cell respiration that converts glucose in food into energy available to cells. Although there is a large amount of LDH in tissue cells, the level in blood is usually very low. If the tissue suffered a loss, more LDH is released into the bloodstream [40]. Serum LDH was tested lasting for 24 h after TBI in order to determine cellular LDH leakage caused by brain damage. Levels were obviously reduced in the VX765 group (Figure 2(f)), indicating the protective role of VX765 during the TBI process. Brain caspase-1 activity was also determined at 24 h after TBI. Results showed that TBI-induced increase in activated caspase-1 was obviously reduced in VX765 mice (Figure 2(g)).

### 3.3. Expressions of Pyroptosis-Associated IL-1*β*, IL-18, Caspase-1, and GSDMD

**Figure 3:**
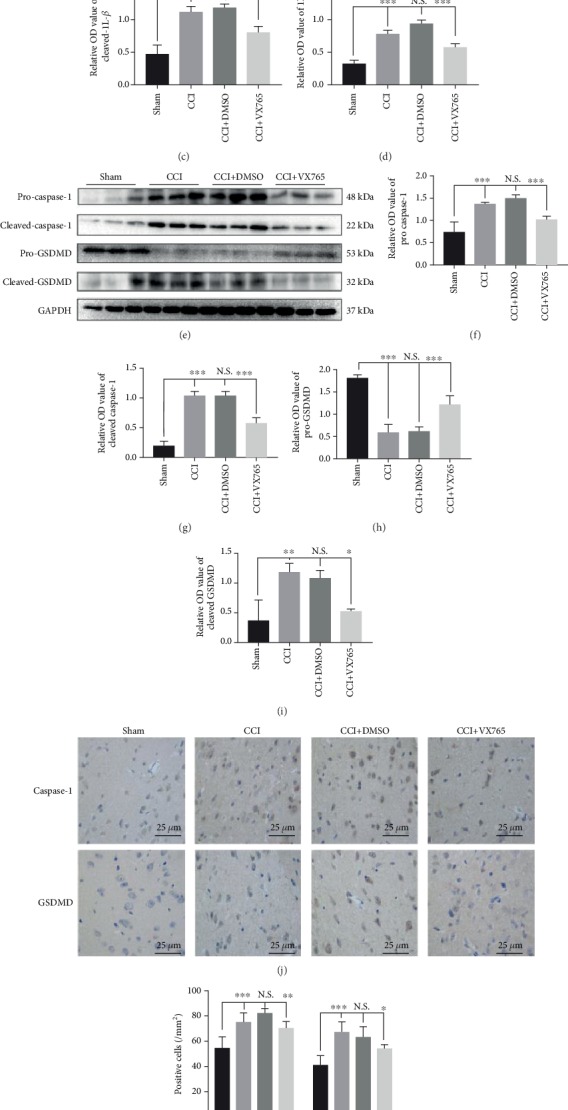
Expression of IL-1*β*, IL-18, caspase-1, and GSDMD (including precursors and actives) associated with pyroptosis in TBI mice. (a) The immunoblot and (b–d) quantitative data of pro-IL-1*β*, cleaved-IL-1*β*, and IL-18 in the injured cortex at 24 h post-CCI. VX765 inhibited the expression of inflammatory factors in the injured cortex. (e) The immunoblot and (f–i) quantitative data of pro-caspase-1, cleaved-caspase-1, and GSDMD in the injured brain at 24 h post-CCI. VX765 suppressed the increased expression of pro-caspase-1 and cleaved-caspase-1 after TBI. It also hindered the cleavage of GSDMD after TBI, characterized by increased expression of pro-GSDMD and decreased expression of cleaved-GSDMD. (j) Immunostaining and (k) quantitative data of caspase-1 and GSDMD in the injured cortex at 24 h post-CCI. As shown immunohistochemically, caspase-1 and GSDMD were distributed in the injured and surrounding areas. The cortex showed a strong increase in caspase-1 and GSDMD staining in the CCI and DMSO groups compared to the VX765 group at 24 h post-TBI. Scale bars: 25 *μ*m. *n* = 3. Data are presented as the mean ± SD. ^∗^*P* < 0.05, ^∗∗^*P* < 0.01, and ^∗∗∗^*P* < 0.001. N.S.: not significant.

In order to decide whether pyroptosis occurred in the mouse TBI model and whether it was affected by VX765 after 24 h, cortical levels of pyroptosis-related proteins IL-1*β*, IL-18, caspase-1, and GSDMD (including precursors and active substances) were evaluated by WB and immunohistochemistry (Figure 3). As shown in Figures 3(a)–3(d), at 24 h after CCI, detection of pro-IL-1*β*, cleaved-IL-1*β*, and IL-18 in the injured cortex revealed that VX765 inhibited the expression of these inflammatory factors. As shown in Figures 3(e)–3(i), VX765 inhibited the increase of pro-caspase-1 as well as cleaved-caspase-1 expression after TBI. In addition, it also blocked GSDMD cleavage after TBI, which had the characteristics of increased expression of pro-GSDMD as well as decreased expression of cleaved-GSDMD. Akin to the WB results, immunohistochemistry showed that caspase-1 and GSDMD were distributed in and around the wound tissue. At 24 h after TBI, cortical tissues in the CCI and DMSO groups showed strong increases in caspase-1 as well as GSDMD compared to the VX765 group (Figures 3(j) and 3(k)). Combined with previous research, these findings confirmed that despite increased expression of pyroptosis-related proteins after the process of TBI, VX765 can inhibit inflammatory responses in the injured cortex by blocking caspase-1 recruitment.

### 3.4. Expressions of NLRs Associated with Pyroptosis

**Figure 4:**
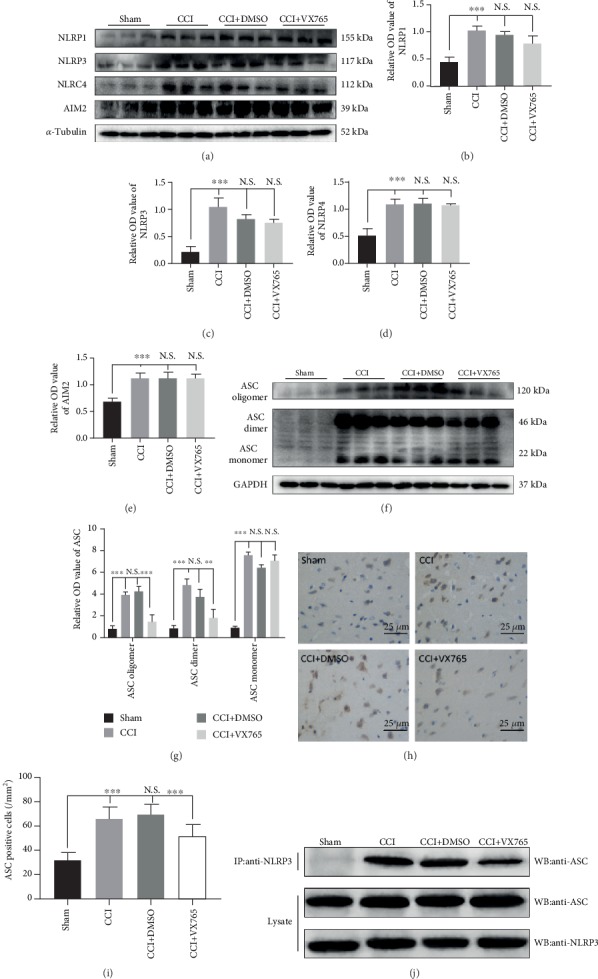
Expression of NLRs, AIM2, and ASC associated with pyroptosis in TBI. (a) The immunoblot and (b–e) quantitative data of NLRs and AIM2 at 24 h post-CCI. Note that the increased expression of NLRs and AIM2 in the injured cortex after TBI was not inhibited by VX765 treatment. (f) The immunoblot and (g) quantitative data of ASC at 24 h post-CCI. The expression of ASC monomers was increased in the injured cortex and was not suppressed by VX765 treatment. In contrast, the increased ASC dimers and oligomers after TBI could be inhibited by VX765 treatment, suggesting that ASC oligomerization was blocked. (h) The immunostaining and (i) quantitative data of ASC in the injured cortex at 24 h post-CCI. As shown immunohistochemically, ASC was distributed in the damaged and surrounding areas. The cortex showed a strong increase in ASC staining in the CCI and DMSO groups compared to the VX765 group at 24 h post-TBI. (j) Co-IP examined the interactions between NLRP3 and ASC and proved that the interactions were significantly enhanced in the CCI and DMSO groups while VX765 treatment inhibited the binding between NLRP3 and ASC. Scale bars: 25 *μ*m. *n* = 3. Data are presented as the mean ± SD. ^∗∗^*P* < 0.01, ^∗∗∗^*P* < 0.001. N.S.: not significant.

To explore the changes in the NLRs, AIM2, and ASC which are closely related to pyroptosis, we examined the expressions of cortical-associated proteins 24 h after TBI with WB and immunohistochemistry. Figures 4(a)–4(e) showed that increased expressions of NLR and AIM2 in the damaged cortex after the process of TBI was not curbed by VX765 treatment. ASC can bridge the inflammasome sensor as well as caspase-1. After the inflammasome is activated, the recruitment of pro-caspase-1 causes supramolecular oligomerization of ASC monomers to form large interwoven fibers (dimer or oligomer), also regarded as ASC-spots or pancakes [41]. ASC-SPECK/imidacloprid corpuscle, as a sign of crawling, plays a very important role in the cleavage of caspase-1 and the release of mature IL-1*β* [42]. Therefore, we also examined cortical expression changes of ASC monomers, dimers, and oligomers upon VX765 administration after the process of TBI. Results showed that expression of ASC monomer in the damaged cortex increased after 24 h, which was not inhibited by VX765 treatment. On the contrary, the increased ASC dimers as well as oligomers after the process of TBI were curbed by VX765 treatment, advising that ASC oligomerization was attenuated (Figures 4(f) and 4(g)). Consistent with the Western blotting assay, immunohistochemical staining for ASC showed a significantly less protein level around the injured cortex in mice treated with VX765 (Figures 4(h) and 4(i)). Later, Co-IP supported that there are strong interactions between NLRP3 and ASC (members of inflammasomes), while this interaction can be restrained by VX765 treatment (Figure 4(j)).

### 3.5. The Impact of Pyroptosis on HMGB1/TLR4/NF-*κ*B Pathway Regulation

**Figure 5:**
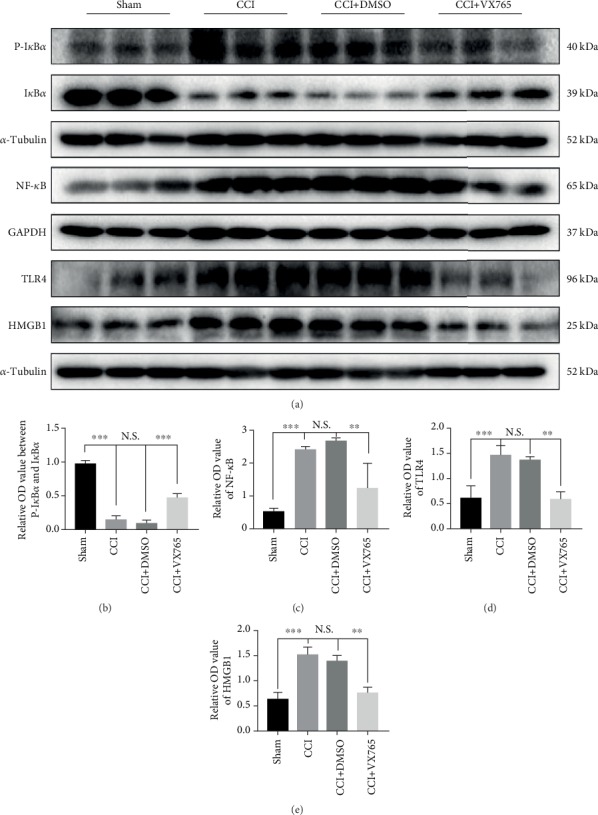
The impact of pyroptosis on regulation of the HMGB1/TLR4/NF-*κ*B pathway on the inflammatory response of the injured cortex after TBI. (a) The immunoblot and (b, c) quantitative data of NF-*κ*B signaling factors in the injured cortex at 24 h post-TBI. VX765 treatment suppressed the activation of NF-*κ*B signaling after TBI by inhibiting the expression of NF-*κ*B and phosphorylated I*κ*B*α* (p-I*κ*B*α*) and promoting that of total I*κ*B*α*. (a) The immunoblot and (d, e) quantitative data of TLR4 and HMGB1 in the injured cortex at 24 h post-CCI. The levels of both were decreased significantly in the VX765 group. *n* = 3. Data are presented as the mean ± SD. ^∗∗^*P* < 0.01, ^∗∗∗^*P* < 0.001. N.S.: not significant.

In order to observe the influence of pyroptosis itself on the TLR4/NF-*κ*B inflammatory response, we examined indicators of the inflammatory pathway by WB after 24 h. We discovered that TLR4 was obviously inhibited in the VX765 group compared with DMSO and CCI groups (Figures 5(a) and 5(d)). Besides, within 24 h after CCI, we quantified the expression changes of NF-*κ*B signal factors in the damaged cortex. NF-*κ*B places downstream of TLR4 and is located in the cytoplasm when it has not been activated. After activation, I*κ*B*α* phosphorylation can be induced. Then, NF-*κ*B is released to the nucleus and thus regulating the release of downstream inflammatory mediators through inflammatory reaction [43]. HMGB1 was recently discovered to be a normal biomarker for TBI as well as cognitive dysfunction, and it is essential for the early prediction and progression of TBI. HMGB1/TLR4 axis is the key element causing neuroinflammation [44], so we also measured HMGB1 levels in the injured cortex 24 h after TBI. We discovered that VX765 curbed the activation of NF-*κ*B signaling after the process of TBI by inhibiting the expression of NF-*κ*B and phosphorylation of I*κ*B*α* (p-I*κ*B*α*) (Figures 5(a)–5(c)). The HMGB1 level was also obviously decreased in the VX765 group (Figures 5(a) and 5(e)) compared with the DMSO and CCI groups. Therefore, inhibition of pyroptosis by VX765 can inhibit the inflammatory response of the damaged cortex after TBI.

### 3.6. Effects of VX765 on Cell Apoptosis and BBB Disruption after TBI

**Figure 6:**
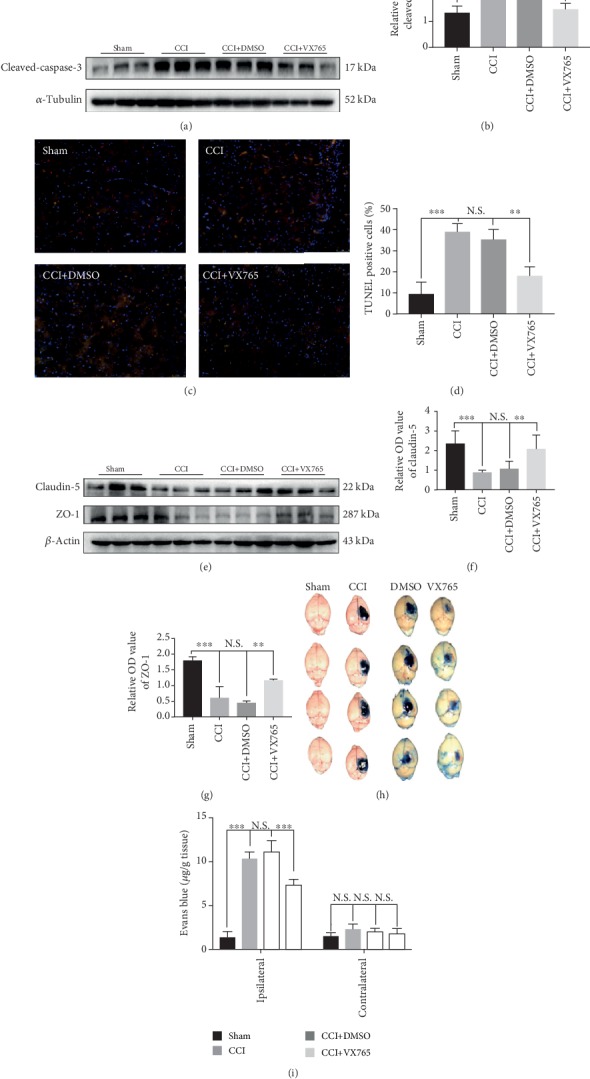
Effect of VX765 on cell apoptosis and BBB disruption after TBI. (a) The immunoblot and (b) quantitative data of cleaved-caspase-3 at 24 h post-CCI in the damaged cortex. Note that the level of cleaved-caspase-3 in the VX765 group was significantly less than that in the CCI and DMSO groups. (c) Counterstaining of TUNEL and Iba-1 and (d) statistical analysis of positive cells were used to detect cortical damage in the TBI model. The apoptosis index (AI, number of TUNEL-positive cells divided by the total cells per field) was calculated. Each AI was assessed from 15 randomly selected fields. Scale bars: 50 *μ*m. *n* = 3. (h) Representative images of EB leakage in CCI groups at 24 h post-TBI. (i) Quantification of EB leakage into the ipsilateral and contralateral hemispheres. VX765 administration significantly decreased EB extravasation in the ipsilateral hemisphere at 24 h after TBI compared with the DMSO and CCI groups. *n* = 4. (e–g) Claudin-5 and ZO-1 expression in the injured cortex was downregulated after TBI according to Western blotting analysis, but VX765 treatment significantly reduced the TBI-induced loss of TJ proteins. *n* = 3. Data are presented as the mean ± SD. ^∗^*P* < 0.05, ^∗∗^*P* < 0.01, and ^∗∗∗^*P* < 0.001. N.S.: not significant.

Previous studies have shown that pyroptosis-associated inflammasomes are involved in apoptosis [45]. Therefore, we investigated the influence of caspase-1 inhibition on apoptosis by WB and TUNEL staining. Through the Western blotting assay, the level of cleaved-caspase-3, which was increased in the cortex after TBI, decreased significantly in the VX765-treated mouse cortex (Figures 6(a) and 6(b)). Consistently, TUNEL-positive cells in the VX765 group were less than those in the DMSO group, and more importantly, these TUNEL-positive cells were also stained by Iba-1 staining, suggesting that the apoptotic cells were microglia (Figures 6(c) and 6(d)). These results demonstrated that VX765 can attenuate caspase-3-mediated apoptosis. Related studies have shown that a posttraumatic inflammation cascade promotes TBI-induced BBB destruction [11]. Therefore, we investigated whether VX765 has a positive effect on pyroptosis-associated inflammatory and inhibitory effect on BBB destruction, and measured BBB permeability by EB dye extravasation. We investigated a significant increase in EB content in the ipsilateral hemisphere of the untreated CCI group compared to the sham group, indicating severe BBB destruction. However, 1 day after TBI, the VX765 treatment group showed significantly less EB extravasation than the DMSO group (Figures 6(h) and 6(i)). Related studies indicate that tight junction (TJ) proteins play major roles in preserving BBB integrity [46]. In order to further investigate the effect of VX765 on TJ proteins after the process of TBI, we used WB to detect the expression of claudin-5 and ZO-1 level 1 day after the injury. Expression levels of both proteins were downregulated in the injured cortex after TBI induction, but these decreases were remarkably reduced by VX765 (Figures 6(e)–6(g)). These results indicate that VX765 administration can significantly alleviate TBI-induced BBB destruction.

### 3.7. Microglial Changes after Trauma

**Figure 7:**
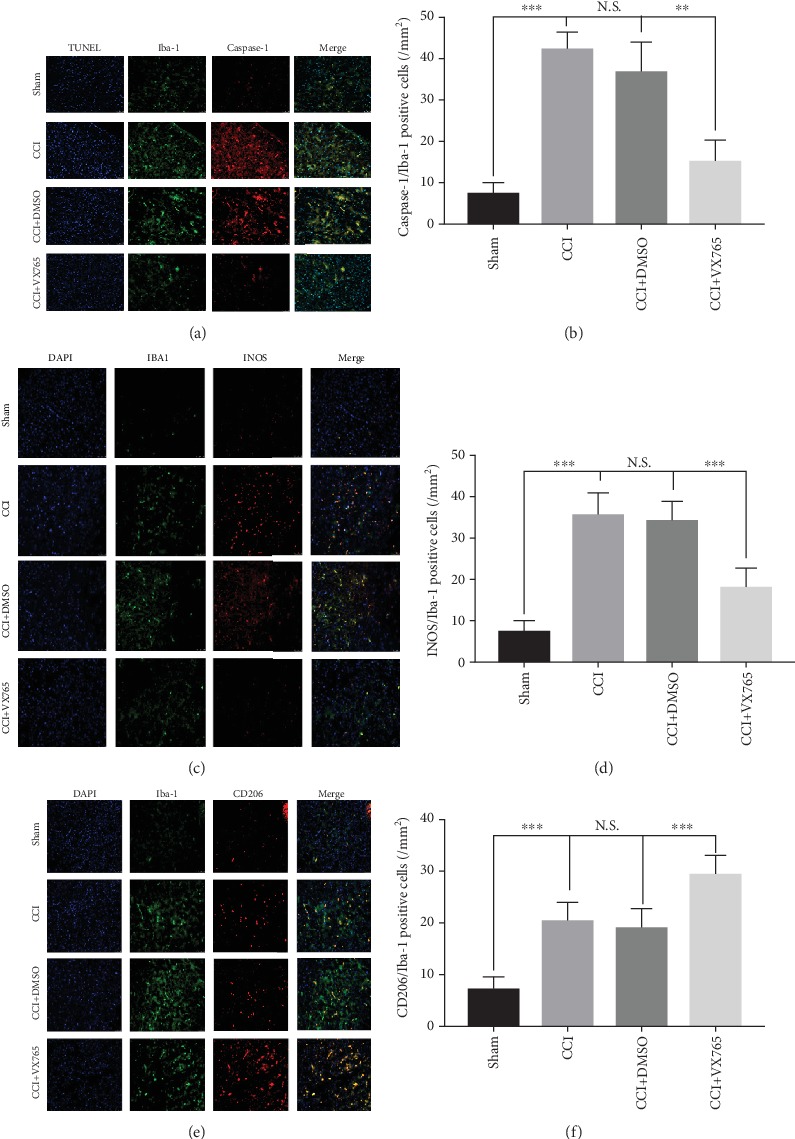
Microglial alterations after trauma. (a) Representative photographs of double immunofluorescence staining for Iba-1 (green), caspase-1 (red), and TUNEL (blue) in the pericontusional cortex at 24 h postinjury. (b) Statistical analysis of immunofluorescence-positive cells. Note that the levels of Iba-1 and caspase-1 in the VX765 group were lower than those in the CCI and DMSO groups. (c–f) Fluorescence expression and proportion of M1 and M2 microglia in the area around the brain injury. (c) Colabeled fluorescent images and (d) proportional statistics of microglia Iba-1 (green) and iNOS (red). Compared with the sham operation group, M1-type microglia (Iba-1 and iNOS double-positive cells) were significantly elevated in the CCI and DMSO groups, and the M1-type microglia decreased significantly in the VX765 group. (e) Colabeled fluorescent images and (f) proportional statistics of microglia Iba-1 (green) and CD206 (red). Compared with the sham operation group, M2-type microglia (Iba-1 and CD206 double-positive cells) slightly increased in the CCI and DMSO groups, and M2-type microglia increased significantly in the VX765 group. Scale bars: 50 *μ*m. *n* = 3. Data are presented as the mean ± SD. ^∗∗∗^*P* < 0.001. N.S.: not significant.

Previous studies have shown that microglia can be activated after trauma, resulting in increased intracellular caspase-1 and pyroptosis [47], and TBI can also promote microglia polarization [37]. To determine whether VX765 treatment affected microglia polarization and intracellular pyroptosis, changes in relevant markers were detected by immunofluorescence. As shown in Figures 7(a) and 7(b), microglia increased and activated after trauma, and intracellular caspase-1 levels and apoptosis rate detected by TUNEL were increased. However, pyroptosis was significantly inhibited by VX765. As shown in Figures 7(c)–7(f), microglial cells are labeled as M1 (iNOS) and M2 (CD 206) phenotypes. TBI obviously increased the expressions of all M1/M2 markers compared with the sham group 24 h after injury. With the aid of VX765 administration, M1-type microglia are transformed into the M2-type, causing less abundance in M1-type cells and more proportion of M2-type cells. These results indicated that VX765 treatment inhibited the pyroptosis-associated inflammatory response in microglia and significantly altered the M1/M2 phenotype balance by curbing M1 activation and enhancing M2 activation after the process of TBI.

### 3.8. The Neuroprotective Effect of VX765 after TBI

**Figure 8:**
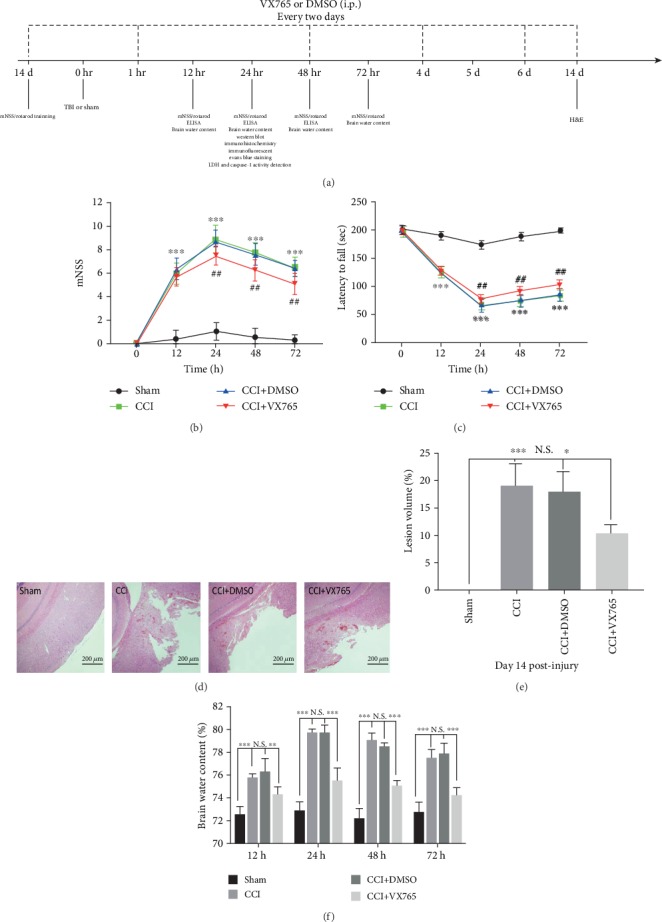
Effect of VX765 on neurological function, brain edema, and cortical lesion size after TBI. (a) Experimental design timeline. i.p.: intraperitoneal injection. (b, c) Neurological recovery was evaluated by (b) mNSS and (c) rotarod tests at 12, 24, 48, and 72 h after TBI. VX765 treatment improved sensory motor and motor functions of mice compared with the DMSO and CCI groups. *n* = 12. ^∗∗∗^*P* < 0.001 vs. sham, ^##^*P* < 0.01 vs. DMSO. (f) Brain water content was evaluated at 24 h postinjury. VX765 treatment reduced the water content at each time point. *n* = 4. (d) Representative H&E staining of brain sections and (e) quantitative analysis of lesion volume at 14 d postinjury. Administration of VX765 significantly reduced the cortical lesion volume of TBI mice on day 14 after surgery compared with the DMSO and CCI groups. Scale bars: 200 *μ*m. *n* = 3. Data are presented as the mean ± SD. ^∗^*P* < 0.05, ^∗∗^*P* < 0.01, and ^∗∗∗^*P* < 0.001. N.S.: not significant.

To investigate whether VX765 still provided neuroprotection after trauma, we measured mNSS, rotarod retention time, and BWC at 12, 24, 48, and 72 h after TBI, together with cortical defect size at day 14 after trauma. The experimental timeline is shown in Figure 8(a). mNSS in the CCI group was obviously higher than that in the sham operation group and reached the peak at 24 h. This value gradually decreased with time. At 12 h, the difference was not obvious (*P* > 0.05) between the CCI group and the VX765 group. However, mNSS in the VX765 group was lower than that in the DMSO group and the CCI group at 24 h, 48 h, and 72 h (Figure 8(b)). In the rotarod test, the sham operation group showed best performance compared to the CCI group. The CCI group had the lowest score for 24 h and then gradually increased. The scores in the VX765 group were higher than those in the DMSO group and CCI group (Figure 8(c)). Through measuring BWC at 12, 24, 48, and 72 h, the influence of VX765 on cerebral edema was further determined. Compared with the CCI group, the treatment of VX765 resulted in obvious reduction of BWC at different time points (Figure 8(f)). We also evaluated the influence of VX765 on brain injury volume on the 14th day after TBI induction. As shown in Figures 8(d) and 8(e), the sham-operated mice showed no serious cortical damage. However, TBI caused significant reduction of brain tissue, and VX765 administration significantly reduced brain tissue. In conclusion, the results proved that VX765 can improve neurological outcomes.

## 4. Discussion

In vivo evidence provided by this study showed that caspase-1 inhibitor, VX765, significantly attenuated TBI-induced brain inflammation as well as neurological deficits. There were three main findings. (1) After TBI, there were significantly more pyroptosis-related inflammasomes in the injured cortex of mice. VX765 could alleviate the expressions of pyroptosis-related mediators around the injured cortex. Microglia participated in the development of pyroptosis 24 h after trauma. (2) VX765 had an inhibitory effect on the HMGB1/TLR4/NF-*κ*B inflammatory pathway as well as weakening effects on pyroptosis and polarization in microglia. (3) VX765 treatment decreased brain edema, tissue loss, inflammatory mediator expressions, BBB destruction, and apoptosis and facilitated the recovery of neurological function after TBI.

Pyroptosis is defined as an inflammatory-mediated and caspase-1-dependent type of programmed cell death, which is only recently discovered. It is stimulated by a series of microbial infections or noninfectious stimuli (e.g., TBI) [15]. The NLR is a recipient that responds to various PAMPs. In NLRs, the signal-specific and functional roles of NLRC4 and particularly NLRP3 have led to great interest in the study of inflammasomes and pyroptosis [48]. The NLRP3 inflammatory corpuscle is one of the major components of the innate immune system and participates in the sterile inflammatory response by enhancing expression of caspase-1 as well as IL-1*β* in a TBI environment [23]. Regarding AIM2, studies have shown that AIM2 inflammasome-mediated neuronal apoptosis is an essential cell death mechanism in the central nervous system infection and damage [49]. AIM2 and NLRP3 act as sensors to take part in the activation of caspase-1, which catalyzes neuroinflammation and ptosis [34]. Our outcomes predict that NLRP1, NLRP3, NLRC4, and AIM2 inflammasomes are activated in the damaged cortex 24 h after CCI. This finding advances our understanding of the distribution of NLR and AIM2 in different cell types in the brain.

Pyroptosis is driven by noncanonical and canonical inflammasomes [34]. In the course of pyroptosis, TLR and/or IFN-mediated initiation upregulates guanylate-binding protein expression in bacterial vacuolar lysis and/or PAMPs (including lipopolysaccharide and bacterial DNA exposure), as well as the corresponding sensors and cytokine precursors [50]. The ability of inflammatory caspase to advance cell lysis includes the rupture of GSDMD in order to advance membrane pore formation [19]. Membrane destruction results in death and reduction of drooping lysed cells and proinflammatory cytokines [17]. Atypical inflammasomes also control NLRP3 classical inflammasome-mediated caspase-1 activation by cleavage of pannexin-1 as well as GSDMD in a cellular intrinsic manner including potassium efflux [51]. Besides, previous researches have expressed that pyroptosis generally induced the release of cytoplasmic LDH [52]. In conclusion, by increasing caspase-1 activity, NLR and AIM2 inflammasomes, GSDMD expression, and blood LDH release, pyroptosis is included in the acute phase of TBI. After administration of the caspase-1 inhibitor VX765, caspase-1 deficiency leads to decreased pyroptosis in the acute phase, less LDH release, reduced neuroinflammation, and inhibition of neurological deficits and microglia death.

NF-*κ*B is one of the most well-known inflammation-related transcription elements produced by almost all eukaryotic cells. It regulates a large number of genes and signaling pathways implied in inflammation [53]. NF-*κ*B has been extensively researched in cellular immune response to infection and inflammatory response as well as autoimmune pathology [54]. Previous studies have shown that certain TLR subtypes are widely demonstrated in the brain and play major roles in regulating inflammation in response to brain damage [27]. TLR4, in particular, is an important mediator in the neuroinflammatory cascade caused by TBI [55]. NF-*κ*B is downstream of TLR4 and is located in the cytoplasm when it is not activated. After activation, I*κ*B*α* phosphorylation was induced. Therefore, NF-*κ*B was released into the nucleus and then regulated the release of downstream inflammatory mediators through an inflammatory response [43]. Previous reports indicate that mRNA levels of HMGB1 are significantly increased in spinal cord injury mice [56]. Recently, studies have reported that HMGB1 is a normal biomarker of TBI, neuroinflammation, epileptogenesis, and cognitive dysfunction and plays a major role in the early prediction as well as progression of TBI [44]. The HMGB1/TLR4 axis is an essential initiator of neuroinflammation [44]. HMGB1 promotes expression levels of NLRP3 as well as caspase-8 inflammasome by activating the NF-*κ*B pathway in the acute glaucoma [57]. Garcia et al. reported that melatonin inhibits NF-*κ*B/NLRP3 activation by regulating the retinoic acid receptor-related orphan receptor *α* pathway [58]. Another group demonstrated that NF-*κ*B increases GSDMD transcription through binding to two proximal binding sites upstream of the gene's promoter field [50]. Therefore, the HMGB1/TLR4/NF-*κ*B pathway is closely associated with the pyroptosis. It also has been mentioned that neuronal death and neurological deficits are closely associated with inflammatory response in TBI [59]. In this study, the results indicated that VX765 can dampen the inflammatory response by attenuating the HMGB1/TLR4/NF-*κ*B signaling pathway in a mouse TBI model. Moreover, the value of mNSS was decreased by VX765 treatment after 24 h of TBI. Therefore, we confirmed the potential role of VX765 in neurological recovery and pyroptosis.

Liu et al. reported the discovery of NLRP3 inflammatory corpuscles in neurons, microglia, and astrocytes; however, other researches emphasized that NLRP3 is primarily expressed in microglia [60]. With the application of double immunofluorescence staining, we determined that microglia were the major source of caspase-1 inflammasome expression at 24 h after trauma. Activated microglia is the main executors of the inflammatory process in the central nervous system and can be polarized into a harmful phenotype (M1) or a favorable phenotype (M2) depending on the microenvironment of its host tissue [61]. Transformation into beneficial M2 microglia can inhibit neuroinflammation and improve central nervous system repair [62]. However, TBI-induced fragile brain microenvironment changes the transient M2 phenotype to the persistent M1 phenotype in white and gray matter [63, 64]. We found that VX765 treatment significantly inhibited microglial activation and recruitment and altered polarization by inhibiting and promoting M1 and M2 activation, respectively. This may be associated with the decrease of proinflammatory cytokines and the increase of anti-inflammatory cytokines [65]. In conclusion, we demonstrated that VX765 regulated the polarization direction of microglia and the severity of pyroptosis by changing the expression of pro- and anti-inflammatory cytokines.

Brain trauma leads to cell damage and BBB destruction. Microglia residing in central nervous system and peripheral immune cells are immediately activated and infiltrated into the injured site. As a result, inflammatory brain injury was caused by the production and release of inflammatory mediators [66]. It is an essential method to reduce BBB injury after TBI in order to reduce secondary brain injury and improve TBI prognosis [67]. In the process of neuroinflammation, IL-1*β* has a profound effect on increases in other proinflammatory cytokines, stimulation and recruitment of microglia and leukocytes, damage of endothelial cell TJ proteins to destroy the BBB, and elicitation of apoptosis [66, 68]. In the present study, VX765, an inhibitor of caspase-1, ameliorated apoptosis evidenced by the decreased expression of cleaved-caspase-3 and declined apoptosis rate. Once apoptotic caspases with long prodomains, termed initiator caspases, were activated, initiators could activate effector caspases including caspase-3, leading to execution of cell death through cleaving substrates. Besides, caspase-1 is considered as the protease able to activate proinflammatory cytokines pro-IL-1*β* and pro-IL-18 which were essential for apoptosis [69]. Along with decreases in caspase-1 and IL-1*β* levels, VX765 administration significantly inhibited BBB destruction and apoptosis after trauma.

VX765 is a potent, selective caspase-1 inhibitor that inhibits the expressions of pyroptosis-associated inflammasomes and IL-1*β* as well as IL-18 production and attenuates pyroptosis in vitro [33, 34]. Previous researches have shown that VX765 inhibits the expressions as well as the activities of NLRP3 and caspase-1 pathways in the lung [35]. Others have reported that VX765 can prevent bone erosion and reduce serum cytokine levels in arthritis [70]. It was recently shown that NLRP3 inflammasomes participate in stress-induced depression by regulating IL-1*β* production in serum as well as in the hippocampus. VX765 also effectively promotes depressive symptoms in mice [71]. The inhibition of caspase-1 by VX765 may be on account of negative feedback on the activation step of the NLRP3 inflammasome or activation of signaling pathways involved in other priming steps, such as interfering with NLRP3 inflammasomes [72]. Therefore, the potential mechanism underlying inhibitive effects of VX765 on pyroptosis-associated inflammatory protein expression needs further study and clarification. Since we used previously reported doses and dosing times, the effects of VX765 may be limited by dose and duration of administration [33, 35]. We found that VX765 treatment also decreased the severity of acute brain edema in mice after TBI. Its mechanism of action is still unclear, so further study is needed to explain this phenomenon. Besides, we observed that VX765 significantly promoted neurological recovery. This suggests that VX765 may be a possible candidate for future TBI clinical trials. Our data indicated the regulation of pyroptosis-associated inflammatory corpuscle activation and other inflammatory pathways in microglia after TBI. TBI induces TLR4 activation on the cell membrane and increases the expression of the downstream NF-*κ*B inflammatory pathway, resulting in the release of IL-1*β* and IL-18 precursors. TBI also induces inflammasome activation and ASC spot formation associated with pyroptosis, resulting in the cleavage of inflammatory factors (pro-IL-1*β* as well as pro-IL-18) derived from the NF-*κ*B pathway into mature biologically active forms. Caspase-1 also cleaves GSDMD, leading to the formation of GSDMD pores. The GSDMD pore-permeable membrane allows secretion of mature cytokines into the extracellular space. Neuroprotection against TBI is achieved by improving the secretion of these inflammatory factors, inhibiting apoptosis, and then protecting the BBB. However, this study has some inadequacy. We selected the protective doses from previous publications without conducting a dose-response study [33, 35]. So as to promote the clinical relevance of VX765 for TBI, further research is needed to decide the optimal dose, administration frequency, and treatment window as well as related potential mechanisms of action. It also manifests the limitations in this study. The improvement of VX765 on neurological impairment was not sufficiently elucidated, which would be further stated in our future studies.

## 5. Conclusion

In conclusion, VX765 has neuroprotective effects against TBI in mice and improves functional recovery after TBI. This may be due to inhibition of activation of the HMGB1/TLR4/NF-*κ*B inflammatory pathway as well as reduction of pyroptosis. As a result, VX765 may be a promising method to prevent or reduce inflammation and nerve injury after TBI.

## Data Availability

Data generated and/or analyzed during the current study can be obtained from the corresponding author on reasonable request.

## Abbreviations

TBI: Traumatic brain injury
CCI: Controlled cortical impact
BBB: Blood-brain barrier
NLRs: NOD-like receptors
AIM2: Absent in melanoma 2
IL-1*β*: Interleukin-1*β*
IL-18: Interleukin-18
GSDMD: Gasdermin D
ASC: Apoptosis-associated spot-like protein
HMGB1/TLR4/NF-*κ*B: High-mobility group box-1/Toll-like receptor 4/nuclear factor-*κ*B
PAMPs: Pathogen-associated molecular patterns
DMSO: Dimethyl sulfoxide
RIPA: Radioimmunoprecipitation assay
WB: Western blotting
LDH: Lactate dehydrogenase
ELISA: Enzyme-linked immunosorbent assay
TUNEL: Terminal deoxynucleotidyl transferase-mediated dUTP-biotin nick end labeling
BWC: Brain water content
EB: Evans Blue
Mnss: Modified neurological severity score.
